# A Fibre‐ vs. cereal grain‐based diet: Which is better for horse welfare? Effects on intestinal permeability, muscle characteristics and oxidative status in horses reared for meat production

**DOI:** 10.1111/jpn.13643

**Published:** 2021-09-22

**Authors:** Federica Raspa, Francesca Rita Dinardo, Ingrid Vervuert, Domenico Bergero, Maria Teresa Bottero, Daniele Pattono, Alessandra Dalmasso, Marica Vinassa, Ermenegildo Valvassori, Elena Bruno, Pasquale De Palo, Emanuela Valle

**Affiliations:** ^1^ Department of Veterinary Sciences University of Turin Grugliasco Italy; ^2^ Department of Veterinary Medicine University of Bari Valenzano Italy; ^3^ Institute of Animal Nutrition, Nutrition Diseases and Dietetics Faculty of Veterinary Medicine Leipzig University Leipzig Germany; ^4^ Public Veterinary Service, ASL TO5 Chieri Italy; ^5^ Public Veterinary Service, ASL CN2 Alba Italy

**Keywords:** horse, intestinal permeability, *Longissimus thoracis et lumborum* muscle, nutrition, oxidative status, welfare

## Abstract

Horses reared for meat production are fed high amounts of cereal grains in comparison with horses raised for other purposes. Such feeding practice may lead to risk of poor welfare consequences. The aim of this study was to investigate the effects of two feeding practices on selected metabolic parameters and production aspects. Nineteen Bardigiano horses, 14.3 ± 0.7 months of age, were randomly assigned to two groups—one fed with high amounts of cereal grains (HCG; *n* = 9; 43% hay plus 57% cereal grain‐based pelleted feed) vs. one fed with high amounts of fibre (HFG; *n* = 10; 70% hay plus 30% pelleted fibrous feed)—for 129 days. At slaught on abattoir, biological and tissue samples were collected to evaluate the microbiological contamination of mesenteric lymph nodes and liver; selected meat quality traits (chemical composition and fatty acid profile of the *Longissimus thoracis et lumborum* muscle); and the oxidative status of the horse. A linear mixed model was used: dietary treatment and sex were fixed effects and their interaction analysed on production and metabolic parameters as dependent variables. Results showed an increased intestinal permeability in the horses fed HCG compared to HFG, according to the significant increased total mesophilic aerobic bacteria counts in mesenteric lymph nodes (*p* = 0.04) and liver samples (*p* = 0.05). Horses in HCG showed increased muscle pH (*p* = 0.02), lighter muscle colour (L) (*p* = 0.01), increased intramuscular fat concentrations (*p* = 0.03), increased muscle glutathione peroxidase and superoxide dismutase activities (*p* = 0.01 and *p* = 0.03, respectively). Moreover, horses in HCG had lower muscle water holding capacity at interaction with sex (*p* = 0.03, lower in female), lower muscle protein content (*p* = 0.01), lower concentration of muscle PUFAs (*p* = 0.05) and lower plasma catalase activities (*p* = 0.05). Our results showed that feeding a high cereal grains diet can have global effects on horse physiology, and thus represents a threat for their welfare.

## 1 INTRODUCTION

Animal welfare is a complex and multidimensional concept. The feeding practice adopted for horses can affect the welfare of these animals through their direct effects on the animals' health as well as by influencing horse behaviour (Lesimple, 2020). Accordingly, horses are grazing animals, adapted to eating forages. Thus, a fibre‐based diet should represent the basis of horse nutrition, respecting the innate herbivorous nature of these animals (Davidson & Harris, 2007). Forages are high in structural carbohydrates and provide at least 50%–70% of a horse's energy requirements through the metabolism of volatile fatty acids (VFA) produced by bacterial fermentation in the hindgut (Merritt & Julliand, 2013). However, due to the demands placed on horses for competitions and/or productive performances (i.e. sport horses and horses destined to meat production), they are often fed with high amounts of energy‐dense feedstuffs rich in hydrolysable carbohydrates, such as starch and simple sugars (Julliand et al., 2006; Raspa, Tarantola, Bergero, Bellino, et al., 2020; Raspa, Tarantola, Bergero, Nery, et al., 2020; Williamson et al., 2011). A number of studies concerning equine nutrition state that starch consumption should be limited to no more than 2 g starch/kg bodyweight (BW)/meal (Durham, 2009; Geor & Harris, 2007; Julliand et al., 2006). Feeding horses with diets characterised by a high starch content can negatively affect their welfare, increasing the risk for gastrointestinal disorders such as colic and gastric ulcers (Durham, 2009; Hudson et al., 2001). In particular, when it reaches the hindgut, the high starch content of a cereal grain‐based diet causes microbiome alterations, leading to an increase in lactic acid production and a drop in pH with subsequent acidosis (Geor & Harris, 2007; Merritt & Julliand, 2013). Acidosis is reported to cause severe damage to the intestinal epithelium, leading to hyperpermeability—also known as ‘leaky gut’ (Stewart et al., 2017). Alterations in intestinal permeability can also lead to the translocation of enteric bacteria and/or their products from the gut lumen into the mesenteric lymph nodes and the portal circulation (Davis et al., 2003; Stewart et al., 2017), with the potential for systemic consequences. A high cereal grain intake has also been associated with several muscular disorders, such as exertional rhabdomyolysis and polysaccharide storage myopathy (PSSM), shown to result from excessive glycogen storage within the muscle (MacLeay et al., 1999; Valberg et al., 1999). Moreover, the ingestion of excessive amounts of rapidly fermentable carbohydrates has been associated with the condition of oxidative stress in horses, and biomarkers of oxidative stress have been proposed as indicators of animal welfare (Celi & Gabai, 2015).

Among the various animal species reared for meat production, also horses reared for this purpose are fed high amounts of cereal grains as a fundamental energy source (Cappai et al., 2013; Lorenzo et al., 2014; Raspa, Tarantola, Bergero, Bellino, et al., 2020; Raspa, Tarantola, Bergero, Nery, et al., 2020). Most scientific studies on the subject report that farms breeding horses for meat mainly rear young horses (Tateo et al., 2008) and that feeding regimes, which include hay plus high amount of cereals (7–8 kg/horse/day; Franco et al., 2013; Lorenzo et al., 2014; Raspa, Tarantola, Bergero, Bellino, et al., 2020; Raspa, Tarantola, Bergero, Nery, et al., 2020; Sarriés & Beriain, 2005) are primarily geared towards fattening the horses.

On such a basis, in view of the fact that nutrition can impact both on animal health and welfare, the aim of the present study was to compare the effects of two different feeding regimes—high cereal grains vs. high fibre—on production and metabolic parameters.

For these reasons, microbiological contamination of mesenteric lymph nodes and liver as potential indicators of altered intestinal permeability have been investigated by two microbiological criteria (Total Mesophilic Aerobic Bacteria counts [TMABc] and Enterobacteriaceae counts) and tested for the presence of pathogenic bacteria (*Salmonella* spp. and *Escherichia coli*). Moreover, selected meat quality traits (chemical composition and the fatty acid profile of *Longissimus thoracis et lumborum* muscle) were evaluated. Finally, horses were investigated for oxidative status by means of antioxidant enzymes and oxidation end‐products determined in different biological fluids and tissues.

## 2 MATERIALS AND METHODS

The present study was approved by the Ethical Committee of the Department of Veterinary Sciences of the University of Turin (Italy, Prot. n. 2202/2019). The study was carried out on the biggest horse farm in Northern Italy, which rears horses with the specific intention of fattening them for meat production. The housing and management features of this farm have previously been described in recent papers published by Raspa, Tarantola, Bergero, Bellino, et al., (2020); Raspa, Tarantola, Bergero, Nery, et al., (2020).

### 2.1 Animals and stable features

Nineteen horses of the Bardigiano breed (12 females and 7 males) aged 14.3 ± 0.7 months (mean ± standard deviation, SD) were treated against internal parasites (1.29 g/100 kg BW; Equalan duo; Merial Animal Health) upon arrival at the farm. During the subsequent 2 weeks, horses were kept together in an outdoor dry lot and fed the same grass hay containing mainly *Lolium Italicum* which was provided ad libitum. After the adaptation period, horses were housed in group pens in a barn with two open sides and no access to any outdoor paddock area. Horses were randomly divided into two group pens (7 x 9 m), which assured a space allowance of at least 6 m^2^ per animal. The group pens were located side by side, each of which was enclosed by horizontal metal rail bars, delimiting the pens at the feed bunk level. Each pen contained a single automatic drinker providing tap water. One flake of fresh barley straw bedding was distributed across over the permanent bedding once a day before the evening meal by means of an automatic straw‐dispersing tractor. Animals were weighed at the beginning and at the end of the trial in order to calculate the average daily gain in bodyweight. All horses were weighed at the same time of the day when they arrived on the farm and the evening before slaughter after the evening meal.

### 2.2 Diets

The animals were randomly assigned to the two groups and they received the same hay (described in Table 1) but a different concentrate feed. One group of horses was individually fed with a high starch and sugar cereal grain‐based complementary feed (HCG; 43% hay plus 57% cereal grain‐based pelleted feed); the other group was individually fed with a fibre‐rich complementary feed (HFG; 70% hay plus 30% pelleted fibrous feed). The composition of the different complementary feed used is provided in Table 1.

**Table 1:** Chemical composition (% as fed) of hay and pelleted feed

	Hay	Cereal grain‐based pelleted feed HCG^a^	Pelleted fibrous feed HFG^b^
DM^c^	89.81	89.91	90.59
Crude protein	6.62	14.21	19.77
Ether extract	1.03	3.69	5.06
Crude fibre	30.04	4.44	11.53
Ash	6.23	8.30	10.78
Starch	0.27	49.50	19.11
NDF^d^	55.20	17.62	27.10
ADF^e^	35.06	6.44	15.28
ADL^f^	4.01	0.73	1.98

^a^High cereal grains group (*n* = 9).

^b^High fibre group (*n* = 10).

^c^Dry matter.

^d^Neutral detergent fibre.

^e^Acid detergent fibre.

^f^Acid detergent lignin.

For the HCG (5 females and 4 stallions), the amount of the complementary feed used was gradually increased over a time: for the first 13 days, they received 3 kg/animal/day, followed by 4.5 kg/animal/day for the subsequent 6 days, and 5 kg/animal/day for a further 36 days; during the final part of the trial, the animals were fed 8 kg/animal/day until the end of the fattening period (72 days). Those quantities were decided by the breeder according to his conventional management system adopted in his farm (Tables 1 and 2).

**Table 2:** Overall nutritional composition of the diets (referred to the total daily diet: hay plus pelleted feed) as fed to the high cereal grains group (HCG) and the high fibre group (HFG) during the fattening period (72 days)

Nutritional components	HCG^a^	HFG^b^
Kg hay/animal/day	6	8
Kg pelleted feed/animal/day	8	3.5
Forage intake/kg BW (%)	1.73	2.32
DM intake (kg)	12.60	10.25
Net energy (MJ)^c^	95.88	53.58
Crude protein (g)	1557.20	1159.60
Digestible Crude Protein (g MADC)	1177.66	723.25
Crude fat (g)	285.40	192.70
Fat contribution to total energy content provided (%)	8.39	10.14
Calcium (g)	377.80	108.22
Phosphorous (g)	188.60	35.79
Lysine (g)	48	76.50
Vitamin E (mg)	399.68	1105
Selenium (mg)	0.48	1.72

^a^High cereal grains group (*n* = 9).

^b^High fibre group (*n* = 10).

^c^Net energy was calculated according to Martin‐rosset, 2015.

For the HFG (7 females and 3 stallions), horses were fed the pelleted fibrous feed which was gradually increased over a time: 1 kg/animal/day for 7 days, 2 kg/animal/day for 9 days, 2.5 kg/animal/day for 25 days, 3 kg/animal/day for 9 days, and finally 3.5 kg/animal/day until the end of the fattening period (72 days). Those quantities were decided by the researchers according to the nutritional requirements of horses as suggested by the French Institute National de la Recherche Agronomique (INRA) (Martin‐Rosset, 2015; Tables 1 and 2). The complementary feed was individually supplied to the horses twice a day (07:00 and 18:00). At the same time, hay was provided and the hay consumption was estimated to be fed 6 kg/animal/day for the HCG and 8 kg/animal/day for the HFG.

Feeds were weighed before each provision to horses and left over were monitored throughout the duration of the trial.

### 2.3 Slaughter procedures and sample collection

At the end of the fattening period (day 129), all animals were slaughtered. The commercial authorised abattoir was 7 km from the horse farm and took less than 25 min travelling time to reach. All the procedures carried out during this phase were supervised by the official veterinarian and conducted according to the European Union regulations (EU Regulation 2009/853 and EU Regulation 627/2019). After slaughtering, selected biological samples were collected as listed below.

Blood samples were collected from the jugular vein by venipuncture into tubes containing EDTA and transported to the laboratory within one hour. Blood plasma was separated by centrifugation at 1500 *g* for 10 min. Aliquots were stored at −20°C for the subsequent analysis of antioxidant enzymes and oxidation end‐products as described below in Section 2.4.

Liver tissue and mesenteric lymph nodes were aseptically collected from the packed viscera immediately after evisceration by a trained operator and placed into sterile bags. Samples were transported to the laboratory at 4°C for microbiological analysis and processed within one hour. A 100 g liver sample was frozen at −20°C for subsequent analysis of antioxidant enzymes and oxidation end‐products as described in Section 2.4. A 100 g liver sample and 100 g of mesenteric lymph nodes were immediately processed to assess their microbiological contamination, as described below in Section 2.5.

The *Longissimus thoracis et lumborum* muscle of the right half‐carcass was immediately refrigerated at 4°C and sampled at the 17/18th thoracic vertebrae level after 24 h of storing at low temperature. One sample was processed for the analyses of muscle characteristics as described below in Sub Section 2.6.1; and one aliquot was stored at −20°C until the subsequent analysis of its chemical composition and fatty acid profile as described below in Sub Sections 2.6.2 and 2.6.3, respectively.

### 2.4 Analysis of antioxidant enzymes and oxidation end‐products

Plasma, liver and muscle samples were analysed for the following antioxidant enzymes: glutathione peroxidase (GPx), catalase (CAT), superoxide dismutase (SOD), according to the methods described by Tufarelli et al. (2016) and Tateo et al. (2020). The following oxidation end‐products were also determined in plasma and muscle samples: thiobarbituric acid reactive substances (TBARS), lipid hydroperoxides (HY) and dinitrophenylhydrazine (DNPH) as carbonylated proteins (PC), according to the methods described by De Palo et al. (2018).

#### 2.4.1 Analysis of thiobarbituric acid reactive substances (TBARs), protein carbonyls and hydroperoxides in plasma

Thiobarbituric acid reactive substances (TBARs) were measured fluorometrically according to Gondim et al., (2009), by adding 100 ml plasma to a 0.37% thiobarbituric acid solution. Plasma reactive carbonyl derivative (RCD) levels were measured according to Faure & Lafond (1995). RCD levels were determined by carbonyl reagent DNPH. Plasma (200 ml) was mixed with 1 ml water and 2 ml 20% trichloroacetic acid and centrifuged at 1000× *g* for 10 min. The pellet was resuspended in 1 ml of 10 mmol/L DNPH and incubated for 60 min at 37.8°C. In the control condition, 1 ml of 1 mol/L hydrochloric acid was used instead of DNPH. Subsequently, 1 ml of 20% trichloroacetic acid was added, and the sample was centrifuged at 1000× *g* for 10 min. The pellet was washed with 1:1 ethanolethyl acetate solution and centrifuged at 1000× *g* for 10 min. The pellet was mixed with 1 ml of 6 mol/L guanidine (diluted in 20 mmol/L dihydrogenphosphate at pH 2.3). Finally, the sample was incubated for 40 min at 37.8°C. The absorbance was measured at 380 nm. Hydroperoxides were analysed according to (Södergren et al., 1998). Aliquots (90 ml) of plasma were transferred into eight microcentrifuge vials (1.5 ml). Ten microliters of 10 mmol/L TPP in methanol were added to four of the vials to reduce ROOHs, thereby generating a quadruplicate of blanks. Methanol (10 ml) was added to the remaining four vials to produce a quadruplicate of test samples. All vials were then vortexed and incubated at room temperature for 30 min prior to the addition of 900 ml of FOX2 reagent. After mixing, the samples were incubated at room temperature for 30 min. The vials were centrifuged at 2400× *g* for 10 min with a swing‐out rotor (Hettich Rotenta/RP centrifuge, Hettich‐Zentrifuge). Absorbance of the supernatant was measured at 560 nm using an Ultraspec 2000 spectrophotometer (Pharmacia Biotech). ROOH concentration in the plasma samples was calculated using the mean absorbance difference between quadruplicates of test samples and blank samples.

#### 2.4.2 Muscle thiobarbituric acid reactive substances (TBARs), protein carbonyls and hydroperoxides analyses

Minced muscle samples (5 g) were placed in a 50 ml test tube and homogenised with 15 ml deionised distilled water (DDW). Samples were treated as described by Maggiolino et al. (2020). The concentration of TBARS was calculated by comparison against a standard curve constructed using 1,1,3,3‐tetramethoxypropane, and the concentration of lipid oxidation was expressed as milligrams of malondialdehyde (MDA) per kg of meat. Two milliliters of homogenate (previously prepared for TBARS determination) was used for hydroperoxide quantification as described by De Palo, Maggiolino et al. (2014); De Palo, Tateo et al. (2014). Results were expressed in micromoles per gram. Meat samples (2 g) were homogenised in 20 ml of 0.15 mol/L KCl for 2 min and analysed for the quantification of protein carbonyls as described by De Palo et al. (2013a).

### 2.5 Procedures to assess microbiological contamination of mesenteric lymph nodes and liver samples

Mesenteric lymph nodes were processed as described by Webb et al. (2017) and Mainar‐Jaime et al. (2013). Accordingly, samples of mesenteric lymph nodes were aseptically trimmed to remove excess fat and fascia. The trimmed lymph nodes were submerged into boiling water for 3–5 s and then flamed using a Bunsen burner for 3 s. Then, they were sterile cut and weighed to obtain 25 g/animal for the detection of *Salmonella* spp., and 10 g/animal for the detection of *E*. *coli*.

Liver samples were surfaced flamed before proceeding with deep subsampling. Liver subsamples were then obtained using a sterile scalpel by cutting deep into the organ's tissue. Samples weighing 25 g/animal and 10 g/animal were used for the detection of *Salmonella* spp. and *E*. *coli*, respectively. Subsequently, samples were homogenised according to the analyses described in the subsequent sections.

#### 2.5.1 Total mesophilic aerobic bacteria counts and Enterobacteriaceae counts

ISO procedures were used for TMABc and Enterobacteriaceae counts (ISO 4833–1:2013 and ISO 21528–2:2017, respectively). Briefly, for the detection of TMAB, tissue samples were diluted in Buffered Peptone Water (BPW; CM 509 B, Oxoid) and appropriately plated onto Plate Count Agar (PCA CM 0325 Oxoid), then incubated at 31°C for 48 h. For the detection of Enterobacteriaceae, Violet Red Bile Glucose Agar (VRBG agar CM 0485 Oxoid, Rodano, Milan) was streaked and incubated at 37°C for 48 h. The results are expressed in CFU/g.

#### 2.5.2 Isolation of *Salmonella* spp

The isolation of *Salmonella* spp. was carried out in accordance with ISO 6579–1:2017. After pre‐enrichment in BPW for 24 h at 37°C, 1 and 0.1 ml of each pre‐enrichment solution was inoculated into 10 ml of Selenite Cystine Broth base (CM 0699, Oxoid) and 10 ml of Rappaport‐Vassiliadis Broth (CM 669 B, Oxoid), respectively, and then incubated at either 37°C (Selenite Cystine Broth) or 41℃ (Rappaport‐Vassiliadis Broth) for 24 h and plated onto selective Xylose Lysine Deoxycholate (XLD) Agar (CM 0469, Oxoid) and Hektoen Enteric Agar (HEA) (CM 0419, Oxoid). Following 24 h incubation, suspect colonies of *Salmonella* spp. were tested by inoculation into Kligler iron agar (CM0033, Oxoid).

#### 2.5.3 Isolation of *Escherichia coli*

The isolation of *E*. *coli* spp. was performed as described in ISO 16649–12:2001 using tryptone bile x‐glucuronide (TBX) medium (Oxoid Ltd). Plates were incubated at 41℃ per 24 h. Suspected colonies of *E*. *coli* spp. were then tested using API 20 Enterobacteriaceae (API 20E) strips (BioMérieux).

### 2.6 Analysis of *Longissumus thoracis et lumborum* muscle samples

#### 2.6.1 Muscle characteristics

Forty‐eight hours after slaughtering, the rheological characteristics of muscle samples were assessed. pH measurement was performed using a portable pH meter with a glass electrode shaped to facilitate meat penetration (Carlo Erba pH 710; Carlo Erba Reagenti). Before each measurement, the pH meter was automatically calibrated for muscle temperature and using pH 4 and pH 7 buffered solutions (Crison).

The colour of *Longissimus thoracis et lumborum* muscle samples was determined according to the CIE (Comission Internationale de l'Eclairage) colour system. A Minolta CR‐300 colorimeter (light source D65; Minolta Camera Co. Ltd.) was used according to the method described by De Palo et al. (2015). Forty‐eight hours after slaughtering, measurements were performed on fresh samples (L a b) and then on thawed samples (L* a* b*) in three different points. At each point, measurements were performed in triplicate, making a total of nine measurements per sample, according to the method described by De Palo et al. (2017). The colorimeter was calibrated according to the Hunter‐lab colour space system using a white title (L* = 99.2, a* = 1.0, b* = 1.9). The a* and b* values were used to determine chroma = (a^2^ + b^2^)^1/2^ and hue (°) = tan−1(b/a) according to De Palo et al., 2012. Water holding capacity, thawing losses and cooking losses were measured as described by De Palo, Maggiolino et al. (2014); De Palo, Tateo et al. (2014). The concentration of haem pigment was determined according to Hornsey (1956). Results are presented as µg of acid haematin/g of muscle wet weight.

#### 2.6.2 Chemical composition

After thawing, samples of *Longissimus thoracis et lumborum* muscle were placed in an oven at 105°C until a constant weight was reached in order to determine moisture content. The protein content was measured according to ISO 937:1978. Intramuscular fat (IMF) was measured according to ISO 1443:1973. Each muscle was homogenised in a chloroform:ethanol solution (1:2, vol/vol) prior to the extraction of total lipids from IMF, performed using the method described by De Palo et al. (2016). Ash content was calculated according to ISO 936:1998.

#### 2.6.3 Fatty acid profile

According to the methods described by De Palo et al. (2015, 2016), fatty acid methyl esters (FAME) were prepared by transesterification using methanol in the presence of 3% hydrochloric acid in methanol (vol/vol). FAME were determined using a Trace GC Thermo Quest Gas Chromatograph (Thermo Electron, Rodano) equipped with a flame ionisation detector. The derivatives were separated on a capillary column (Supelco SP‐2380 fused‐silica column, 120 m length, 0.25 mm internal diameter and 0.20 mm film thickness). The injector and the detector temperatures were held at 260°C. Column oven program temperatures were as follows: T1 = 80°C, hold 1 min; T2 = 150°C, ramp at 15°C/min, hold 2 min; T3 = 220°C, ramp at 5°C/min, hold 2 min; T4 = 250°C, ramp at 15°C/min, hold 5 min. The flow rate of the carrier gas (He) was set at 0.8 ml/min. FAME identifications were based on the retention times of reference compounds (Sigma‐Aldrich) and mass spectrometry. Fatty acid composition was expressed as the percentage of total FAME.

The amount of saturated fatty acids (SFA), unsaturated fatty acids (UFA), monounsaturated fatty acids (MUFA), polyunsaturated fatty acids (PUFA), n–3 and n–6 fatty acids, SFA/UFA, SFA/MUFA and SFA/PUFA were calculated to assess nutritional implications. Finally, atherogenic and thrombogenic indices were calculated according to the formulas provided by De Palo et al. (2017):

Atherogenic index (AI) = (C12:0 + 4 x C14:0 + C16:0) / [ΣMUFA + ΣPUFA (n–6) and (n–3)]

Thrombogenic index (TI) = (C14:0 + C16:0 + C:18) / [0.5ΣMUFA + 0.5ΣPUFA (n–6) +3ΣPUFA(n–3) + (n–6)/(n–3)]

### 2.7 Statistical analysis

Data were statistically analysed using the software JMPpro v15 (SAS Institute). Each parameter was tested for normal distribution using the Shapiro–Wilk test and normalised, when necessary, by box‐cox transformation. A linear mixed‐effects model was constructed, and the model fixed effects were the dietary treatment, the sex and their interaction. Then, each horse within sex and diet was considered as experimental unit and used as random variable for all analyses. The initial BW was set as a covariate for the slaughter BW model. Least squares means were separated using *T*‐Student's adjusted *p*‐values when at least a tendency F‐test (*p* ≤ 0.10) was detected in the fixed effect interaction term.

## 3 RESULTS

### 3.1 Animals

Table 3 reports the mean (SEM) initial bodyweight (iBW) of the horses of each group upon their arrival at the farm, the mean (SEM) slaughter bodyweight at end of the study (sBW) and the calculated average (SEM) daily bodyweight gain (ADG) for the two groups (HCG and HFG).

**Table 3:** Mean (SEM) initial bodyweight (iBW), mean (SEM) slaughter bodyweight (sBW) at the end of the trial (129 days) and the calculated mean (SEM) daily bodyweight gain (ADG) for the two groups (HCG and HFG)

	HCG^a^		HFG^b^		*p*‐value		
	Female	Male	Female	Male	Diet	Sex	Diet*Sex
iBW	216.6 (4.02)	218.75 (5.44)	222 (2.07)	219 (2.08)	‐	‐	‐
sBW	346.6 (2.42)	349 (4.38)	343.43 (0.92)	346.67 (1.76)	0.14	0.22	0.61
ADG	1.01 (0.03)	1.01 (0.03)	0.94 (0.02)	0.99 (0.02)	0.15	0.20	0.57

^a^High cereal grains group (*n* = 9).

^b^High fibre group (*n* = 10).

No differences in sBW according to diet, sex or their interaction were evident between the two groups of horses at the end of the trial. Moreover, ADG showed no differences in the two groups of horses according to dietary treatment, sex or their interaction.

### 3.2 Microbiological contamination of mesenteric lymph nodes and liver samples

As shown in Table 4, TMABc were found increased in HCG than in HFG for both mesenteric lymph nodes (*p* = 0.04) and liver samples (*p* = 0.05), indicating a different microbial contamination in those tissues according to the dietary treatment. No differences between HCG and HFG were found in mesenteric lymph nodes (*p* = 0.31) and liver samples (*p* = 0.11) for Enterobacteriaceae counts. Moreover, no samples were found to be contaminated by *Salmonella* spp. or *E*. *coli*.

**Table 4:** TMABc (Total mesophilic aerobic bacteria counts) and Enterobacteriaceae counts (HCG vs. HFG): median values (plus 25th–75th percentiles) expressed as CFU/g

		HCG^a^		HFG^b^		*p*‐value		
		Female	Male	Female	Male	Diet	Sex	Diet*Sex
Mesenteric lymph nodes	TMABc	36*10^2^ (7*10^2^–83.75*10^2^)	4*10^2^ (1.75*10^2^–13.75*10^2^)	2*10^2^ (1*10^2^–4*10^2^)	2*10^2^ (1.50*10^2^–2.50*10^2^)	0.04*	0.34	0.09
	Enterobacteriaceae	55 (10–90)	5 (0–10)	10 (0–20)	0 (0–10)	0.19	0.21	0.42
Liver	TMABc	11.50*10^2^ (4*10^2^–127*10^2^)	38.25*10^2^ (4.38*10^2^–70.25*10^2^)	1*10^2^ (1*10^2^–2*10^2^)	1*10^2^ (0–7*10^2^)	0.05*	0.28	0.95
	Enterobacteriaceae	20 (0–55)	25 (2.5–70)	0 (0–10)	0 (0–20)	0.11	0.69	0.85

^a^High cereal grains group (*n* = 9).

^b^High fibre group (*n* = 10).

^*^Statistical significance *p* < 0.05.

### 3.3 Muscle characteristics and chemical composition of Longissimus thoracis et lumborum muscle

Table 5 shows the mean values (SEM) of the muscle characteristics and the chemical composition of the *Longissimus thoracis et lumborum muscle* samples obtained from horses reared using the two different feeding strategies (HCG vs. HFG). The pH was lower in HCG vs. HFG according to diet (*p* = 0.02). Water holding capacity was lower in HCG vs. HFG according to the dietary treatment (*p* = 0.04). Moreover, this latter finding resulted to be affected by the sex of the animals (*p* = 0.03) since *Longissimus thoracis et lumborum muscle* from females in HCG showed lower water holding capacity than that of females in HFG. Moreover, muscle colour in HCG was characterised by increased lightness (*L*) (*p* = 0.01) compared with muscle samples from HFG. Regarding the chemical composition of the muscle, lower moisture content (*p* = 0.03), increased protein content (*p* = 0.01) and increased concentration of intramuscular fat (IMF; (*p* = 0.03) was found in muscle samples from horses in HCG compared with those from HFG according to the dietary treatment. No differences were observed in ash concentration between the two groups.

**Table 5:** Muscle characteristics and chemical composition (HCG vs. HFG)

	HCG^a^		HFG^b^		*p*‐value		
	Female	Male	Female	Male	Diet	Sex	Diet*Sex
pH	6.68 (0.06)	6.70 (0.05)	6.49 (0.07)	6.54 (0.07)	0.02*	0.63	0.85
Water holding capacity (%)	80.27 (0.42)^A^	81.27 (0.81)^AB^	82.37 (0.32)^B^	81.17 (0.08)^AB^	0.04*	0.83	0.03*
Haematin (µg/g)	250.31 (17.88)	236.19 (38.99)	229.87 (26.52)	259.5 (68.66)	0.97	0.83	0.55
L^c^	38.65 (0.58)	39.20 (1.39)	36.23 (0.57)	37.00 (0.28)	0.01*	0.44	0.90
a^d^	16.46 (0.60)	16.65 (0.46)	17.33 (0.22)	16.39 (0.39)	0.50	0.41	0.22
b^e^	−2.46 (0.48)	−1.50 (0.38)	−1.55 (0.18)	−1.04 (0.47)	0.09	0.07	0.56
L*^f^	36.86 (1.29)	37.70 (1.34)	35.98 (0.23)	37.46 (0.28)	0.57	0.24	0.74
a*^g^	15.96 (0.55)	16.75 (0.56)	16.71 (0.35)	16.26 (0.38)	0.71	0.73	0.23
b*^h^	−1.53 (0.34)	−1.01 (0.58)	0.44 (0.17)	−1.20 (0.33)	0.71	0.21	0.88
Moisture (%)	70.44 (0.20)	70.48 (0.51)	71.49 (0.31)	71.63 (0.88)	0.03*	0.84	0.90
Protein (% of DM^i^)	75.86 (1.27)	75.34 (2.11)	79.37 (0.82)	80.23 (1.90)	0.01*	0.91	0.64
IMF^j^ (% of DM)	11.8 (1.92)	13.08 (2.99)	8.31 (0.85)	7.08 (1.58)	0.03*	0.99	0.52
Ash (% of DM)	4.30 (0.32)	4.83 (0.52)	4.94 (0.29)	4.67 (0.52)	0.56	0.76	0.33

Data shown are means (SEM).

A,B Means with different superscripts differ at *p* < 0.05.

^a^High cereal grains group (*n* = 9).

^b^High fibre group (*n* = 10).

^c^Lightness on fresh samples

^d^Redness on fresh samples.

^e^Yellowness on fresh samples.

^f^Lightness after thawing.

^g^Redness after thawing.

^h^Yellowness after thawing.

^i^Dry matter.

^j^Intramuscular fat.

*Statistical significance *p* <0.05.

### 3.4 Fatty acid profile of the *Longissimus thoracis et lumborum* muscle

The fatty acid profiles of muscle samples from horses reared using different feeding regimes (HCG vs. HFG) are reported in Table 6. Muscle from horses fed with high amounts of fibre showed an increased concentration of C20:5 (*p* = 0.03), PUFA (*p* = 0.05) and *n*6 (*p* = 0.04) than muscle from horses fed with high amounts of cereal grains.

**Table 6:** Fatty acid profile (expressed as % of fatty acid methyl esters) of *Longissimus thoracis et lumborum* muscle samples (HCG vs. HFG). Data shown are means (SEM)

	HCG^a^		HFG^b^		*p*‐value		
	Female	Male	Female	Male	Diet	Sex	Diet*Sex
C10:0	0.06 (0.01)	0.05 (0.00)	0.05 (0.01)	0.05 (0.00)	0.25	0.22	0.97
C12:0	0.10 (0.01)	0.11 (0.00)	0.12 (0.01)	0.12 (0.02)	0.24	0.25	0.79
C14:0	2.01 (0.26)	2.30 (0.44)	2.25 (0.44)	1.95 (0.12)	0.70	0.87	0.84
C15:0	0.61 (0.17)	0.53 (0.05)	0.55 (0.06)	0.61 (0.18)	0.76	0.83	0.96
C16:0	28.11(0.69)	27.13 (0.61)	27.05 (0.74)	28.55 (1.40)	0.84	0.77	0.17
C16:1	4.81 (0.27)	4.97 (0.35)	5.00 (0.27)	5.37 (0.37)	0.38	0.42	0.74
C17:0	2.94 (0.48)	3.20 (0.63)	4.45 (1.04)	3.08 (1.87)	0.53	0.62	0.46
C18:0	7.04 (0.45)	6.58 0.41)	6.76 (0.56)	7.14 (0.63)	0.81	0.94	0.48
C18:1	30.42 (0.41)	30.74 (0.47)	29.42 (0.75)	28.85 (2.17)	0.15	0.90	0.65
C20:0	0.12 (0.00)	0.12 (0.01)	0.12 (0.01)	0.15 (0.00)	0.17	0.14	0.15
C18:2n–6	17.23 (0.88)	18.13 (0.77)	17.67 (0.74)	17.16 (1.76)	0.79	0.85	0.49
C18:3n–6	0.02 (0.00)	0.03 (0.00)	0.03 (0.00)	0.03 (0.01)	0.93	0.96	0.62
C18:3n–3	4.55 (0.11)	4.32 (0.24)	4.52 (0.25)	4.84 (0.23)	0.33	0.87	0.28
C20:4n–6	0.64 (0.08)	0.53 (0.03)	0.66 (0.16)	0.74 (0.11)	0.59	0.82	0.32
C20:5n–3	0.02 (0.00)	0.02 (0.00)	0.07 (0.04)	0.03 (0.01)	0.03*	0.46	0.67
C22:0	0.41 (0.01)	0.41 (0.02)	0.45 (0.02)	0.42 (0.02)	0.26	0.58	0.44
C22:6n–3	0.88 (0.07)	0.88 (0.09)	0.87 (0.07)	0.93 (0.55)	0.81	0.73	0.71
SFA^c^	41.47 (0.52)	40.44 (0.71)	41.80 (0.63)	42.06 (0.89)	0.19	0.60	0.38
UFA^d^	58.57 (0.52)	59.60 (0.70)	58.23 (0.63)	57.96 (0.89)	0.18	0.60	0.37
MUFA^e^	35.23 (0.50)	35.71 (0.52)	34.41 (0.87)	34.23 (2.35)	0.30	0.89	0.76
PUFA^f^	22.57 (0.31)	22.89 (0.36)	24.52 (0.68)	24.90 (2.51)	0.05*	0.71	0.98
n3	5.34 (0.17)	4.98 (0.31)	5.50 (0.32)	5.74 (0.28)	0.32	0.65	0.27
n6	17.03 (0.33)	17.91 (0.06)	19.02 (0.48)	19.16 (2.23)	0.04*	0.49	0.62
n6/n3	3.08 (0.12)	3.62 (0.20)	3.51 (0.18)	3.33 (0.23)	0.75	0.40	0.11
SFA/UFA	0.71 (0.02)	0.68 (0.02)	0.72 (0.02)	0.73 (0.02)	0.15	0.62	0.46
SFA/MUFA	1.18 (0.02)	1.13 (0.02)	1.22 (0.04)	1.24 (0.10)	0.14	0.79	0.50
SFA/PUFA	1.79 (0.08)	1.71 (0.10)	1.77 (0.08)	1.79 (0.12)	0.72	0.75	0.62
AI^g^	24.38 (0.81)	24.92 (1.01)	24.87 (0.92)	24.80 (1.90)	0.87	0.84	0.79
TI^h^	2.11 (0.10)	1.89 (0.06)	2.01 (0.11)	2.26 (0.18)	0.27	0.90	0.07

^a^High cereal grains group (*n* = 9).

^b^High fibre group (*n* = 10).

^c^SFA: saturated fatty acids.

^d^UFA: unsaturated fatty acids.

^e^MUFA: monounsaturated fatty acids.

^f^PUFA: polyunsaturated fatty acids.

^g^AI: atherogenic index.

^h^TI: thrombogenic index.

*Statistical significance *p* < 0.05

### 3.5 Antioxidant enzymes and oxidation end‐products

Table 7 shows the results obtained from oxidative enzyme analyses. Muscular GPx and muscular SOD were higher in samples from HCG compared with those from HFG according to the dietary treatment (*p* = 0.01 and *p* = 0.03), whereas plasma CAT was lower in samples from HCG compared with those from HFG (*p* = 0.05). Of the biochemical metabolites resulting from oxidation pathways (Table 8), higher concentrations of muscular TBARs were evident in samples from HFG compared with samples from HCG (*p* = 0.01).

**Table 7:** Plasma, muscle and hepatic concentrations of glutathione peroxidase (GPx), catalase (CAT) and superoxide dismutase (SOD)

		HCG^a^		HFG^b^		*p*‐value		
		Female	Male	Female	Male	Diet	Sex	Diet*Sex
GPx^c^	Plasma (µmol/mg) Median (25th−75th)	0.07 (0.07–0.07)	0.09 (0.05–0.16)	0.08 (0.05–0.14)	0.12 (0.07–0.14)	0.40	0.36	0.98
	Muscle (U/mg) Median (25th−75th)	0.14 (0.12–0.23)	0.25 (0.15–0.26)	0.12 (0.11–0.14)	0.13 (0.11–0.14)	0.01^*^	0.38	0.47
	Liver (µmol/mg) Mean (SEM)	0.26 (0.01)	0.22 (0.02)	0.22 (0.02)	0.24 (0.02)	0.70	0.77	0.11
CAT	Plasma (µmol/mg) Median (25th−75th)	0.84 (0.63–0.88)	1.04 (0.69–1.31)	1.41 (0.83–1.47)	1.20 (1.01–7.15)	0.05*	0.25	0.99
	Muscle (U/mg) Median (25th−75th)	5.03 (3.13–7.04)	3.34 (2.67–4.57)	2.96 (2.78–3.46)	4.03 (2.47–5.52)	0.50	0.60	0.23
	Liver (µmol/mg) Median (25th−75th)	536.89 (517.84–539.79)	519.7 (513.51–537.14)	522.06 (517.88–523.21)	534.79 (516.15–538.61)	0.84	0.92	0.16
SOD	Plasma (µmol/mg) Median (25th−75th)	15.38 (7.88–19.07)	7.80 (4.97–14.73)	6.55 (6.29–17.93)	7.49 (7.02–14.03)	0.71	0.389	0.44
	Muscle (U/mg) Median (25th−75th)	17.60 (14.68–18.26)	16.69 (15.59–18.33)	16.47 (5.48–17.53)	6.56 (5.91–17.54)	0.03*	0.66	0.61
	Liver (µmol/mg) Mean (SEM)	115.86 (1.98)	112.69 (3.90)	111.36 (2.33)	114.82 (1.80)	0.68	0.96	0.26

^a^High cereal grains group (*n* = 9).

^b^High fibre group (*n* = 10).

^c^Expressed as oxidised NADPH content.

*Statistical significance *p* < 0.05.

**Table 8:** Plasma and muscle concentrations of TBARs, hydroperoxides and carbonylated proteins (HCG vs. HFG)

		HCG^a^		HFG^b^		*p*‐value		
		Female	Male	Female	Male	Diet	Sex	Diet*Sex
TBARs^c^	Plasma (nmol MDA/ml) Mean (SEM)	1.33 (0.12)	1.15 (0.12)	1.33 (0.05)	1.17 (0.13)	0.90	0.11	0.94
	Muscle (mg MDA/kg) Mean (SEM)	0.26 (0.02)	0.36 (0.07)	0.50 (0.06)	0.47 (0.07)	0.01*	0.57	0.31
Hydroperoxides	Plasma (µmol/L) Mean (SEM)	5.25 (0.43)	5.40 (0.58)	5.29 (0.22)	5.73 (0.45)	0.66	0.48	0.72
	Muscle µmol/g Median (25th−75th)	0.46 (0.42–0.54)	0.69 (0.48–0.88)	0.55 (0.45–0.59)	0.5 (0.42–0.66)	0.81	0.23	0.11
Carbonylated proteins^d^	Plasma (µmol/ml) Mean (SEM)	98.85 (3.63)	101.10 (10.18)	94.43 (5.71)	90.67 (15.97)	0.41	0.93	0.73
	Muscle (nmol DNPH/mg) Mean (SEM)	1.25 (0.19)	1.43 (0.13)	1.24 (0.12)	1.15 (0.16)	0.39	0.78	0.41

^a^High cereal grains group (*n* = 9).

^b^High fibre group (*n* = 10).

^c^Expressed as malonaldehyde (MDA) content.

^d^Expressed as dinitrophenylhydrazine (DNPH) content.

*Statistical significance *p* < 0.05.

## 4 DISCUSSION

The present study was carried out under field conditions without any possibility of choosing the horses involved in the trial or to change the breeder's management choices for the HCG. As a consequence, it was not possible to establish isoenergetic or isoproteic diets for the two experimental groups. Accordingly, the higher TMABc in the lymph nodes and liver samples found in the HCG could be a consequence of higher bacterial translocation. Regarding the Enterobacteriaceae counts of the liver samples, although no statistically significant difference was detected between groups, it is interesting to note that whilst Enterobacteriaceae were detected in the liver samples from HCG, the median content in HFG was zero.

A multitude of factors may trigger the intestinal barrier dysfunctions that generate a leaky gut, including infectious diseases, drugs, exercise or heat stress (Lambert, 2009). However, in agreement with Stewart et al. (2017), we can hypothesise that the diet was one of the main factors contributing to the differences between the groups of the present study. In fact, all the horses were healthy and admitted to the slaughterhouse without any clinical signs or the requirement for any medical treatment.

Here, we explored selected traits between groups, focusing on the muscle characteristics and chemical composition of the *Longissimus thoracis et lumborum* muscle. In particular, muscle from female horses in HFG showed a higher water holding capacity; and a higher moisture content and a lower pH were identified according to the dietary treatment. In both groups, muscle pH was found to be higher than the values reported in other studies. For example, Gill (2005) reported the pH of horse muscle to be generally below 6. Similarly, Seong et al. (2017) reported pH values around 5.75, with a significant increase in pH the longer samples had been stored (frozen). The low pH values reported in those studies are likely related to the fact that during the development of rigour mortis, muscle glycogen is converted to lactic acid (Lawrie, 1953). After slaughter, glycolysis continues in tissues until the glycogen substrate is depleted, resulting in the accumulation of acidic glycolytic end‐products and a drop in pH (Muir et al., 1998). Our results suggest the existence of differences in the biochemical pathways (e.g. the glycolytic rate) underway in the muscle between groups. The high pH values detected in the present study could be due to different levels of muscle glycogen compared to the studies previously cited. Unfortunately, it was not possible to measure the muscular glycogen in this present study.

The values of water holding capacity recorded in this study were in agreement with the data reported in the literature on horse meat (De Palo et al., 2013b; Sarriés & Beriain, 2005). The significantly higher mean value found in the HFG samples vs. those from HCG could be due to the lower fat deposition between muscle fibres, the higher protein content and the higher moisture content (Tateo et al., 2008). A previous study found that increasing the requirements up to 200% in Italian Heavy Draft horses (IHDH) did not affect intramuscular fat content or the water holding capacity of muscle (De Palo, Maggiolino, et al., 2014; De Palo, Tateo, et al., 2014; De Palo et al., 2017), but in those studies, a different breed (IHDH) was studied compared the breed used in our study (Bardigiano). Moreover, the present study revealed a significant effect of feeding regime on both these muscle features. It is likely that the difference in results is due to the different characteristics of the feeding trials, which here focussed on different starch to fibre ratios. In addition, the animals fed HFG were fed less protein and less fat and even the mineral composition was also different.

Even if the diets were not isoenergetic and isoproteic, in the authors’ opinion some considerations should be taken into account. Interestingly, no statistical significance between groups was found in slaughter BW and ADG (see Table 3). According to the calculation of the net energy provided to the horses per day, the high cereal grain diet supplied 42.3 MJ more than that provided by the diet characterised by high amounts of fibre. According to the French Institute National de la Recherche Agronomique (INRA), a daily body weight gain of 1 kg/day for a horse weighing 350 kg is possible if the animal is supplied with 14 MJ plus its maintenance requirement (46.1 MJ; Martin‐Rosset, 2015). These findings indicated that the extra energy level supplied with the high cereal grain diet did not result in a significantly higher daily body weight gain compared with that achieved in the horses of HFG. This finding is surprising since horses in HCG were fed more energy than horses in HFG. Anyways, the high cereal grain diet overcomes the starch digestibility of 2 g of starch/kg BW as suggested by some authors (Durham, 2009; Julliand et al., 2006). Not all the estimated energy of the high cereal grain diet was used because the starch level in the diet exceeded the digestive capacity of the horse's intestine (Durham, 2009). Moreover, an additional point that we should consider is that a high cereal grain diet can cause high glycaemic response, resulting in increased reactivity behaviours (Bulmer et al., 2015; Hothersall & Nicol, 2009). Horses in HCG may spent more energy in locomotion/reactivity behaviours than horses in HFG. Both considerations should be taken into account for future studies. In conclusion, the extra energy supplied with the high amounts of cereal grains is counterproductive, both from economic and welfare points of view.

Regarding colorimetric patterns, the fresh muscle samples from the HCG group showed higher lightness values compared with those from HFG, whereas these differences did not exist after thawing. Lightness in muscle is related both to the amount of intramuscular fat and to the water content on the cut surface (Mancini & Hunt, 2005). Colour changes in meat from foals are affected by slaughtering age and post‐thawing time (De Palo et al., 2012). The different IMF values could explain the tendency towards higher lightness values in muscle from HCG compared with that from HFG, both in fresh and in thawed meat. The significant differences in lightness in fresh muscle could be due to the different water holding capacities, whereas, after thawing and post‐thawing water losses, the differences in lightness were not statistically significant. Moreover, muscle colour can also be affected by the fatty acid composition of IMF (Lorenzo et al., 2014); indeed, differences in the fatty acid profiles of the two groups were also revealed here.

The diet is one of the main factors influencing the concentration of IMF in horse muscle (Franco et al., 2013; Lorenzo et al., 2014), and diet can influence the fatty acid profile of IMF (Tateo et al., 2008). In fact, several studies have recently underlined that horse breed, slaughter weight and management practices, including feeding regime, affect the fatty acid composition of horses (Juárez et al., 2009; Lorenzo et al., 2010; Sarriés et al., 2006). However, to the best of our knowledge, no studies have quantified the effects of a feeding regimen based on high amounts of fibre on the fatty acid composition of *Longissimus thoracis et lumborum* muscle of horses. Here, we found that the PUFA concentration was higher in muscle from HFG compared with that from HCG. In particular, this result was related to the higher concentration of n6 PUFAs and n3 eicosapentaenoic acid (EPA, C20:5n–3). These differences likely reflect differences between the two diets supplied. Among raw ingredients of the fibrous pelleted feed oilseeds (flaxseeds and dehulled sunflower seeds) was included at dose of 45 g/day during the final 72 days of the fattening period. Regarding the high cereal grain diet, the fat component was essentially supplied by the maize as a main ingredient. However, the total quantity of fat provided by the two diets was similar (see Table 2; HCG = 285.40 g, HFG = 192.70 g; fat contribution to total energy content: HCG = 8.39%, HFG = 10.14%). Interestingly, although HCG presented a higher IMF concentration, the HFG was characterised by a better fatty acid profile, and this result could provide an important incentive to change the feeding practices of horses reared for meat production (Carrillo et al., 2016).

It has been shown that a higher IMF content results in lower moisture content (Duckett et al., 1993; Reagan et al., 1977). Our data align with the literature since HCG displayed a higher IMF content alongside with lower moisture. The mean moisture content was 70.5% and 71.5% for HCG and HFG muscles samples, respectively, in accordance with previous studies conducted on 11–24 months horses (Juárez et al., 2009; Sarriés & Beriain, 2005; Tateo et al., 2008).

Horse muscle is characterised by a high protein content, which varies according to a number of factors, such as sex, muscle type and production system employed (Lorenzo et al., 2014). The French system (Martin‐Rosset, 2015) reports that for a daily growth of 1 kg BW, the total dietary protein requirements should be 733 g MADC/day for a horse weighing 350 kg (where MADC—Matières Azotèes Digestibles Cheval (MADC)—expresses horse digestible crude protein, which represents the estimated measure of the quality of the absorbed amino acids provided by a diet). According to this, horses in the HFG (with a mean sBW of 344.40 kg) would have needed to consume 692 g MADC/day for an average daily BW gain of 0.96 kg. In this study, the HFG diet provided 723 g MADC/day. On the other hand, horses in the HGC (with a sBW of 347.8 kg) would have needed to consume 735 g MADC/day for a daily BW gain of 1.01 kg. However, the horses in HCG were actually supplied with 1178 g MADC/day.

It is important to note that not only should the protein content of a feed meet the total MADC requirements, but also provide proteins of high biological value. In particular, in horse diets, lysine is the main limiting amino acid, especially if diets are cereal grain‐based (Urschel & Lawrence, 2013). In fact, in our study, the horses in HCG received an estimated 48 g of lysine in the diet. On the contrary, the high fibre group was supplied with 76.50 g of lysine. Therefore, these differences could have affected the development of the muscle.

Regarding oxidative status, the higher concentration of PUFAs in muscle samples from HFG compared with that found in HCG could explain the higher muscular concentration of TBARs in the HFG. In fact, the different oxidative stability of IMF is reported to be related to the saturation index of fatty acids (Mahecha et al., 2009). On the contrary, muscular GPx and muscular SOD were higher in HCG than in HFG. Although higher oxidative stress is related to lower GPx and SOD levels, the higher levels in HCG compared with in HFG remains unexplained. In particular, GPx activities are related to selenium intake, and a low selenium intake is related to low GPx activities and vice versa (Avellini et al., 1999). As shown in Table 2, the horses in HCG received only 400 mg of Vitamin E and 0.48 mg of selenium per day, whereas those in HFG were supplied with 1105 mg of Vitamin E and 1.72 mg of selenium. Selenium and Vitamin E are dietary antioxidants which synergistically support endogenous antioxidant systems to reduce reactive oxygen species damages. Limited data are reported from experimental feeding trials on effective nutritional supplementation in Vitamin E in horse meat. However, taking into account scientific studies carried out on other species (Cardenia et al., 2011; Voljč et al., 2011), the α‐tocopherol levels—natural isoform of the fat‐soluble vitamin E group—in tissues and plasma were significantly influenced by the level of dietary supplementation, leading to higher stability of meat lipids. Moreover, Cappai, Pudda et al., (2020); Cappai, Taras et al., (2020) recommended to monitor the Vitamin E intake in the context of adequate feeding practices for health and welfare assessment. In particular, since α‐tocopherol is synthesised and stored chiefly in the green plants, the same authors suggested that a higher dietary intake of Vitamin E is important in stabled horses when they are fed on hay.

Finally, the higher plasma levels of CAT in the horses belonging to HFG suggest that the animals tended to be protected from oxidative damage, as this enzyme is involved in one of the most rapid and effective systems for reducing oxygen free radicals (Ighodaro & Akinloye, 2018). A high fibre source in the diet can effectively promote antioxidant defence by enhancing the free radical‐scavenging ability of the plasma and other relevant organs (Fang et al., 2017). However, no studies have been carried out to date on the antioxidative effects of dietary fibre intake and different fibre components on horse tissue. Even if in this study group replication was not possible, and it is certainly important, this does not preclude the fact that this study can be a source of important suggestions for further studies.

## 5 CONCLUSIONS

The present study shows that feeding horses high amounts of cereal grains is wasteful from an economic stance and harmful from a welfare point of view. In fact, the high amounts cereal grains in the diet did not result with any difference in daily bodyweight gain or with any positive effect on muscle characteristics. Instead, our results support the notion that feeding horses high amounts of cereal grains can lead to a condition of increased intestinal permeability. We also showed that diet affects the concentrations of GPx, CAT and SOD; although plasma, muscle and liver were characterised by distinct differences. We hope this work will encourage further scientific research to improve the feeding practices used in horses' farms in order to safeguard the welfare of horses reared for meat purposes encouraging adequate education of farmers.

## 6 ANIMAL WELFARE STATEMENT

The authors confirm that the ethical policies of the journal, as noted on the journal's author guidelines page, have been adhered to and the appropriate ethical review committee approval has been received. The authors confirm that they have followed EU standards for the protection of animals used for scientific purposes and feed legislation.

## Data Availability

The data that support the findings of this study are available from the corresponding author upon reasonable request.
